# Estimation of the Number of Subslab Soil Gas Samples to Collect to Characterize Vapor Intrusion Under a Large Building

**DOI:** 10.1155/ina/2860696

**Published:** 2025-08-19

**Authors:** John H. Zimmerman, Alan Williams, Brian Schumacher, Chris Lutes, Rohit Warrier, Laurent Levy, Gwen Buckley, Brian Cosky, Chase Holton, Kate Bronstein

**Affiliations:** 1 US Environmental Protection Agency (EPA), Office of Research and Development (ORD), Center for Environmental Measurement & Modeling (CEMM), Watershed and Ecosystem Characterization Division (WECD), Research Triangle Park, North Carolina, USA; 2 US EPA, ORD, CEMM, Ecosystem Processes Division, Athens, Georgia, USA; 3 Federal and Environmental Solutions, Jacobs Engineering Group Inc., Cary, North Carolina, USA; 4 Center for Environmental Health, Risk, and Sustainability, RTI International, Research Triangle Park, North Carolina, USA; 5 Federal and Environmental Solutions, Jacobs Engineering Group Inc., Boston, Massachusetts, USA; 6 Federal and Environmental Solutions, Jacobs Engineering Group Inc., Virginia Beach, Virginia, USA; 7 Federal and Environmental Solutions, Jacobs Engineering Group Inc., Indianapolis, Indiana, USA; 8 Denver Office, GSI Environmental Inc., Lakewood, Colorado, USA

**Keywords:** spatial variability, subslab soil gas, temporal variability, vapor intrusion

## Abstract

Upward migration of vapors from subsurface contamination into overlying buildings is known as vapor intrusion (VI) and can result in exposure of the building’s inhabitants to contaminants that can cause detrimental health effects. Multiple lines of evidence (MLEs), such as groundwater, soil, soil gas, and indoor air volatile organic compound (VOC) concentrations, are used to evaluate a building for VI and potential risk of occupant exposure. Background sources of contaminants contained within a building can result in a false positive determination of VI and installation of mitigation systems that are not needed. To avoid a false positive determination, some VI guidance documents recommend the prioritization of subslab soil gas (SSSG) concentrations over indoor air concentrations for determination of a VI issue. If the SSSG VOC concentrations are above a determined concentration, then VI is assumed to be possible; depending upon the concentration, immediate mitigation may be required. The major challenge to characterizing VI potential is the number of samples needed to confidently assess VI exposures due to the extreme variability in vapor concentrations across both time and space, and this study explores variability in SSSG and how many SSSG samples are needed. To address this issue, SSSG samples were collected between December 2020 and April 2022 from six commercial buildings in Fairbanks, Alaska, and between May 2019 and June 2021 from a large, compartmentalized warehouse at a coastal site in Virginia. Types of samples collected included indoor air; outdoor air; SSSG; soil gas; radon; differential pressure; indoor and outdoor temperature; heating, ventilation, and air conditioning (HVAC) parameters; and other environmental factors. To illustrate how these results can inform estimates of expected SSSG variability and thus the number of samples required to characterize variability, the temporal and spatial variabilities of the results observed at the test sites were used as a “similar population” to estimate necessary sample sizes for characterization of VI levels and to explore how temporal and spatial factors may influence estimates. The estimated SSSG sample requirement ranged from 1 to 80 samples and thus showed the substantial sensitivity of the systematic project planning equation to cases in which the action level and the average concentration are similar. We recommend that the estimated number of samples generated from the collected data for the buildings should only be used as a starting point for planning purposes. The number of SSSG samples to initially collect at a large building to characterize VI can be calculated, but the actual number should include adjustments for features of a building (e.g., past usage, separate foundations, and footers) and conditions at a site (e.g., proximity to source and depth to groundwater) that may alter the required number of SSSG samples.

## 1. Introduction

Vapor intrusion (VI) is the migration of vapor-forming chemicals from subsurface contaminant sources to the indoor air of overlying buildings [1]. Federal, state, and industry VI guidance documents generally recommend following a multiple lines of evidence (MLE) approach when evaluating occupant risk and determining whether mitigation is necessary. The MLE approach often involves collection of groundwater, soil, soil gas, or indoor air samples to evaluate the “VI pathway,” along with screening level comparisons or more complex data analysis and modeling. Soil gas and indoor air concentration results are key lines of evidence for determining whether volatile organic compounds (VOCs) have migrated near, below, or into buildings. Indoor air concentration results are often heavily weighted in decision-making, but these results can be subject to contributions from background sources of VOCs, and temporal variability of 1 to more than 2 orders of magnitude has been reported in residential [2, 3] and commercial or industrial settings [4, 5].

Some VI guidance documents emphasize soil gas over indoor air sampling results in VI pathway interpretation and decision-making [6, 7]; however, current guidance provides limited direction regarding subslab soil gas (SSSG) sampling. In the absence of recommendations in federal guidance, environmental professionals often reference recommendations found in state-level guidance [7, 8] or develop site-specific sampling plans based on their interpretation of the conceptual site model (CSM). The number of SSSG sampling locations recommended for characterizing soil gas below small structures (< 150 m^2^; ~1600 ft^2^), such as residential buildings, is generally two or three locations per building (one per 50 to 75 m^2^); however, the minimum number recommended for larger buildings is less clear, with recommended values ranging from 5 to 12 locations per building based on the size of the building footprint [8]. For example, the New Jersey Department of Environmental Protection (NJDEP) VI Guidance [8] Section 3.3.1.4 recommends two SSSG sampling points in buildings with a footprint of < 139 m^2^ (1500 ft^2^) with the recommended number of SSSG sampling points increasing with the square footage of the building footprint. Federal and other state-level VI guidance documents are less specific about SSSG sampling point density but generally emphasize more samples in larger buildings.

Soil gas concentrations are subject to significant temporal and spatial variability [9–13], both of which can delay site decision-making. For example, SSSG concentrations collected monthly over 12 months at Naval Air Station Lemoore (California) varied by a factor of 4 [13]. Similarly, SSSG samples collected over six events in 12 months at six residential buildings at Lowry Air Force Base (Colorado) exhibited concentration variability of up to 2 orders of magnitude [2]. Variations of up to two orders of magnitude in SSSG concentrations were also reported in two sampling-intensive studies conducted over periods exceeding 1 year at a residential duplex in Indianapolis, Indiana [14]; a residential home in Layton, Utah [10, 11]; and an industrial building in coastal Virginia [12, 15]. In general, SSSG concentration patterns from the sampling-intensive studies were characterized by long-term stability or changes that occurred gradually over weeks to several months. These gradual changes in temporal and spatial variabilities at a site required a longer data collection period to clarify emerging trends within the data, thereby delaying site decision-making.

VOC concentrations in soil gas change over time because of changes in equilibrium partitioning conditions between the vapor phase, aqueous phase (i.e., soil moisture), soil matrix, and nonaqueous phase liquids (NAPLs) [16, 17]. These changes in equilibrium partitioning can result from changes in subsurface temperature or moisture conditions or may be related to other meteorological factors that vary over time. The temporal variability of soil gas concentrations has been attributed to seasonal and weather variations, along with activities in overlying buildings that pressurize or depressurize the building envelope, with the greatest variation observed in samples collected close to the soil surface [1, 11, 17, 18]. One hypothesis is that precipitation and snow or ice cover can displace soil gas or create surface capping effects [2]. Likewise, wind or barometric pressure changes can contribute to the spatial displacement of soil gas [2, 9, 19]. Diurnal and seasonal variations of subsurface temperature or changes in differential temperature between the overlying structure and the soil may also affect the distribution of SSSG through the stack effect [20]. Recent work has highlighted the importance of climate zone and the location of the source relative to the building in controlling the magnitude of observed variability [20–23].

This study focuses on the spatial and temporal variabilities of SSSG VOC concentrations at six buildings in Alaska and one warehouse-sized building in Virginia. The following research questions are explored in this paper: (1) How do temporal and spatial trends in SSSG VOC concentrations vary across commercial buildings and sites? (2) How many SSSG samples are sufficient to predict indoor air risk and impact risk management decisions at a building?

## 2. Site Descriptions

The primary site is in Fairbanks, Alaska, in a commercial area with a diverse mix of small, medium, and large buildings for residential, commercial, and government use. Two chlorinated solvent (tetrachloroethylene [PCE] and trichloroethylene [TCE]) plumes, referred to as the Eastern and Western Gaffney Road plumes (Figures S1a and S1b), resulted from separate historical dry cleaner operations in the area. Figure S1a shows the two plumes (divided by Cushman Street near the source) and displays how they migrate northwest toward mixed-use neighborhoods. Six buildings with varying characteristics (Table 1) overlying these two plumes were selected for this study including the following:

- Not for profit (overlying eastern plume source area).
- Tailor and tuxedo rental business (overlying eastern plume source area).
- Residential-style office (overlying eastern plume).
- Government building (overlying eastern plume).
- Office building (overlying western plume).
- Church (overlying western plume).

Since the initial discovery of groundwater contamination by chlorinated VOCs in 1993 [24], several site investigations have been performed at these and other buildings [25–27]. A summary of historical sampling results and other site information is provided in the supporting information (Table S1 and Figure S1b). The second site is in coastal Virginia, where soil and groundwater contamination (TCE and PCE) have been under investigation since 1995 [12]. Additional method details related to “Virginia Site A” are provided in prior publications [12, 28].

## 3. Experimental Methods

### 3.1. SSSG Probe Construction.

Eight Vapor Pin-style SSSG probes were installed inside the church at several locations shown in Figures S2 through S7 [29]. Leak testing at each new and historic location was performed using the water dam method [30]. Water dam leak checks passed at all probe locations. At Virginia Site A, several styles of SSSG sampling probes (i.e., Vapor Pin, traditional subslab, and California style) were installed at 19 closely spaced locations within a hexagon-shaped sampling grid, and two more California-style probes [28] were installed: one in a supply room and one in a restroom.

### 3.2. SSSG VOCs.

Soil gas VOCs were determined using a modified EPA Compendium Method TO-17 [31]. A measured volume of soil gas was pulled through a sorbent tube using a calibrated syringe. Stainless steel thermal desorption tubes with dimensions of 8.9 cm (3.5 in.) in length by 0.64 cm (0.25 in.) in outside diameter, packed with approximately 0.2 g of Tenax TA, were used for sample collection. Analysis was accomplished by heating the sorbent tube and sweeping the desorbed compounds onto a secondary “cold” trap for water management and analyte refocusing. The secondary trap was rapidly heated for efficient transfer of compounds and subsequent separation by gas chromatograph (GC) and detection by mass spectrometry (MS).

Continuous VOC data were collected at the church and residential-style office using two separate GC/electron capture detector (ECD) instruments. The indoor and outdoor air, SSSG, and exterior soil gas monitoring locations at each building are summarized in Table S3 and shown on Figures S2 through S7. The sample introduction systems were configured with two separate multiport valves on each instrument. Indoor and outdoor air samples were collected through the primary valve (A) and soil gas through the secondary valve (B) to limit cross-contamination potential for each matrix. Teflon tubing was connected from the various sampling locations to the GC/ECD instruments. Vacuum pumps pulled samples through the tubing and into the instruments’ sample loops before analysis.

The GC/ECD instruments achieved a reporting limit of 0.1 part per billion by volume (ppbv) or 0.7 *μ*g/m^3^ for PCE. In the case of indoor air PCE, estimating the instrument’s method detection limit based on a 3:1 signal-to-noise ratio yielded a value of about 0.02 ppbv, which is equivalent to about 0.14 *μ*g/m^3^ for PCE. Indoor and outdoor air samples (Table S3) were analyzed every 2–3 h. Soil gas samples (Table S3) were analyzed every 6–13 h, producing two to three samples per day.

At the Virginia Site A building, continuous TCE concentration data were collected throughout the building from May 2019 to June 2021 using a GC/ECD instrument. At Fairbanks, discrete VOC data were collected at all six commercial buildings from December 2020 to March 2022, with additional continuous GC/ECD VOC data collected at the church and residential-style office between August 2021 and March 2022.

Meteorological measurements at both sites were made using on-site weather stations and supplemented with data from the nearest National Weather Service facility. Additional details on the methods and devices used for meteorological measurements are provided in Figures S8 and S9.

## 4. Results and Discussion

### 4.1. Degree of Temporal and Spatial Variabilities Across All Buildings and Locations.

**Figure 1:**
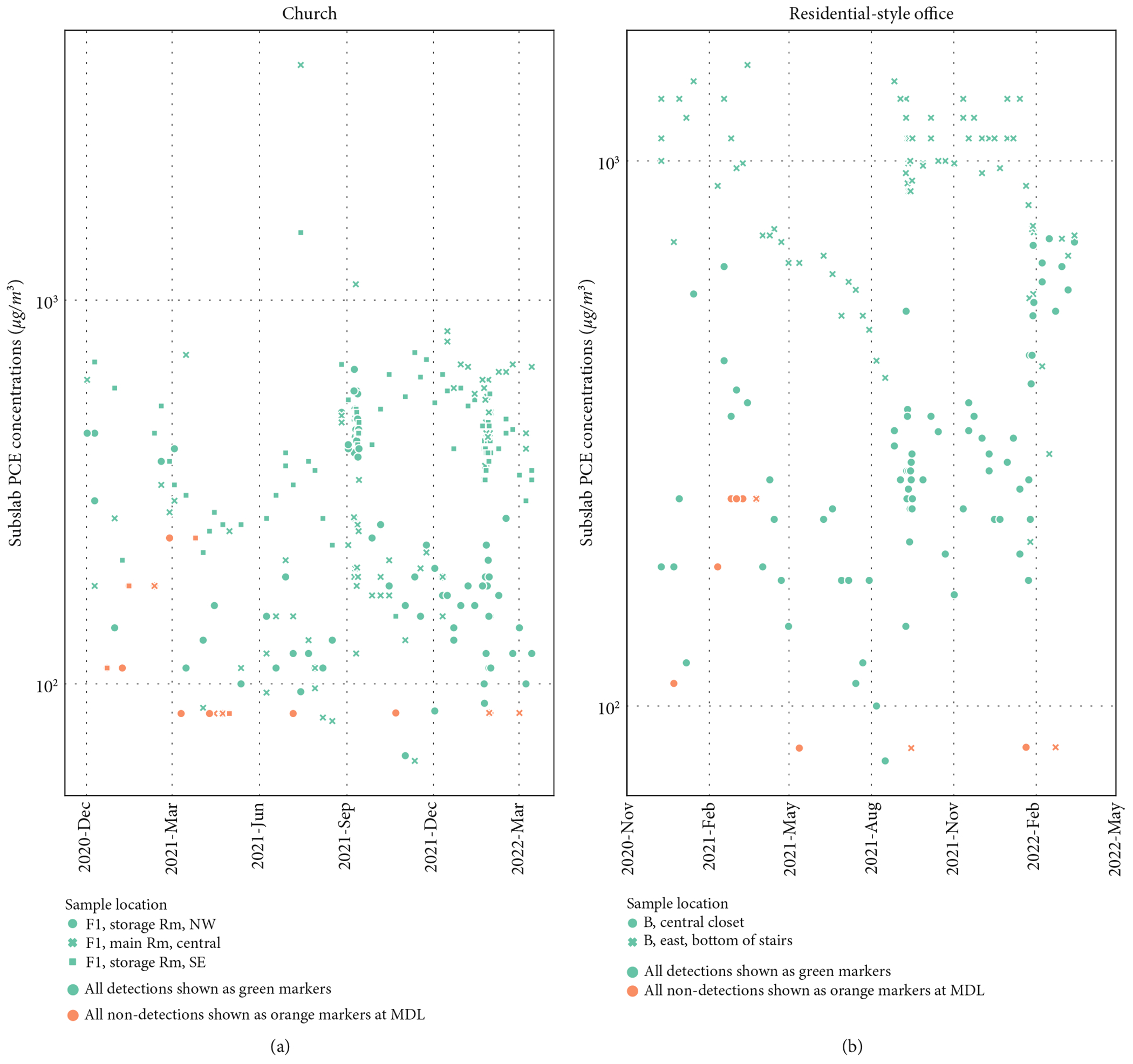

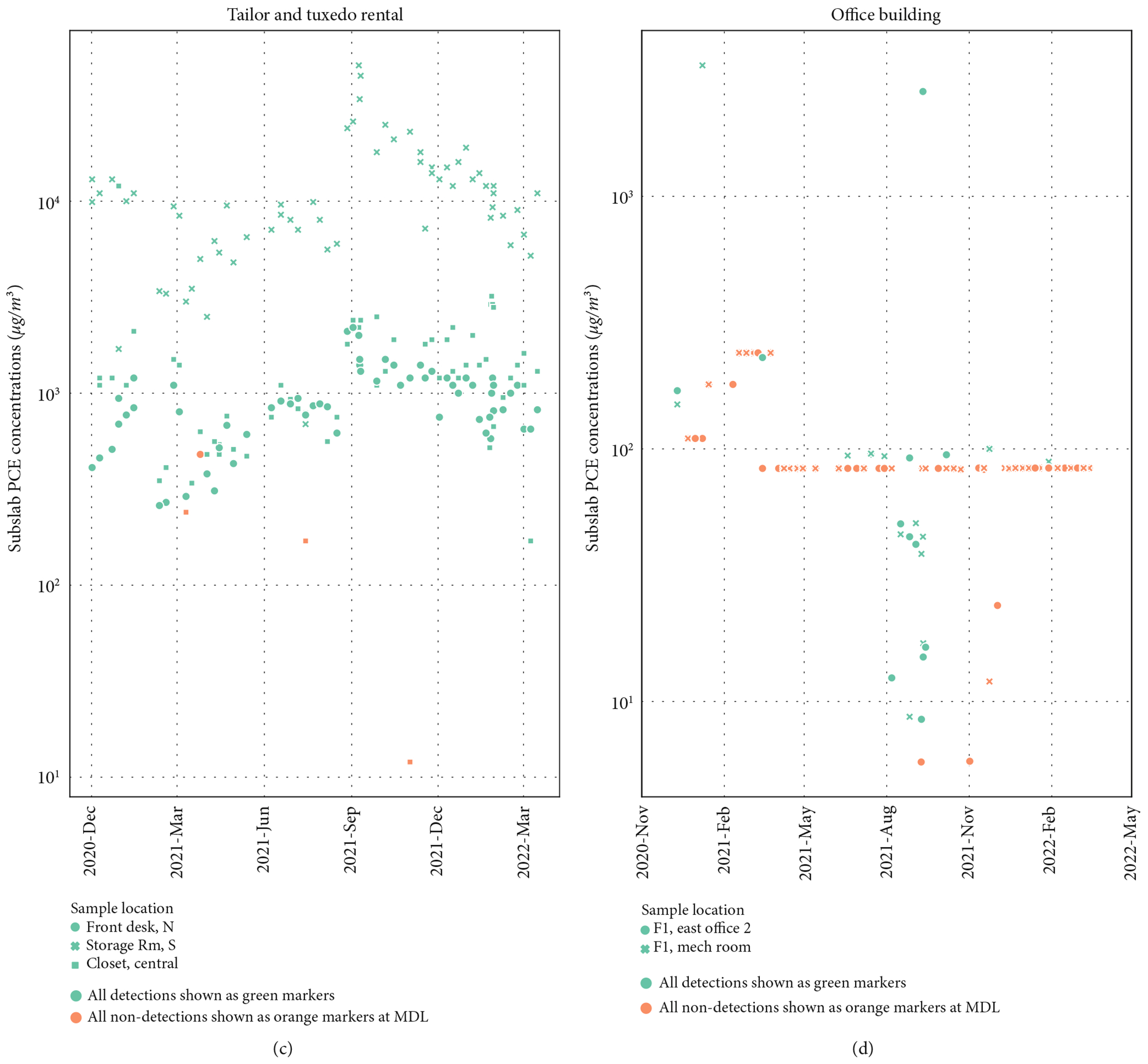

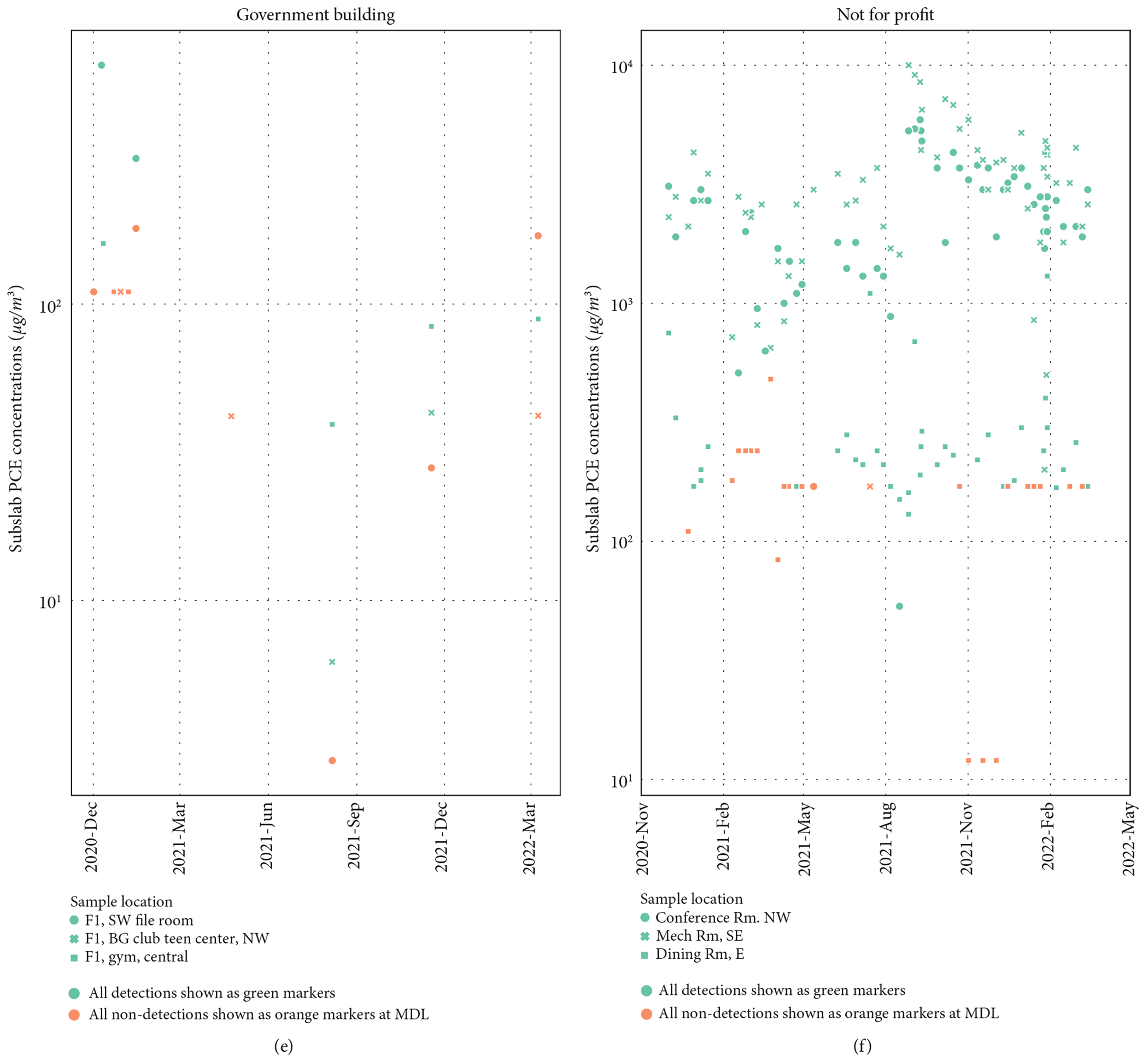
(a–f) PCE concentration versus time in Fairbanks SSSG ports, TO-17 samples from December 2020 to April 2022 for the six buildings studied.

**Figure 2:**
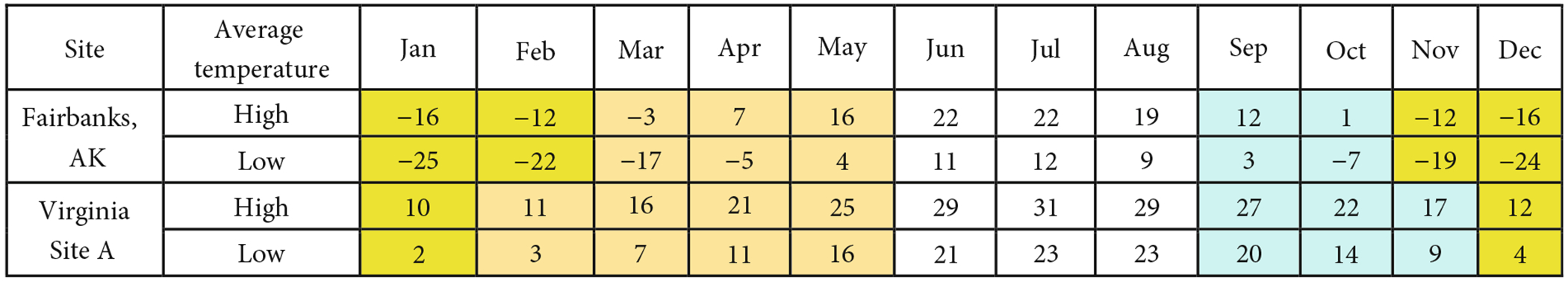
Average monthly ambient temperatures (°C) at the Fairbanks, Alaska, and Virginia sites [33, 34]. Note: Yellow cells are winter, peach is spring, white is summer, and aqua is autumn.

SSSG concentrations at the Fairbanks site spanned four orders of magnitude across space and time, whereas results at Virginia Site A spanned over seven orders of magnitude. As discussed in previous studies conducted at Virginia Site A [12, 28], limited temporal variability was observed in SSSG locations over a 24-month study period; although multiple events were documented that resulted in significant changes in SSSG VOC concentrations, they did not follow a clear seasonal pattern. Changes in SSSG concentrations were attributed to variation in soil moisture and wind direction [12, 32]. Spatial variability between SSSG sampling locations was more significant at Virginia Site A, with additional hot spots discovered with increasing sampling location density. In contrast, at the Fairbanks site, temporal variability generally corresponded with seasonal VI drivers (Figure 1). The timing of seasons at the site in Fairbanks is considerably different than the seasonal timings at the Virginia site (Figure 2). In Fairbanks, winter can last for 4 months, November through February, whereas winter at the Virginia site occurs in December through January (yellow cells in Figure 2). Spring in Fairbanks usually occurs from March to May, and spring at the Virginia site is the months of February through May (yellow cells in Figure 2). The summer in Fairbanks and the Virginia sites occur during the months of June, July, and August (white cells in Figure 2). Fall in Fairbanks is during September and October, whereas in Virginia, it is September through November (see aqua cells in Figure 2).

The wide difference in concentration ranges measured between the two sites reflects differences in vapor source strength and lateral distance from the source. The greatest temporal and spatial variabilities were observed at the Virginia Site A building (a rectangle approximately 60 × 180m [200 × 600 ft]), which directly overlies historical soil and shallow groundwater sources [12, 28]. Conversely, the buildings at the Fairbanks site overlie relatively dilute and stable groundwater plumes or adjoin a weaker source with a thicker vadose zone (4–4.6 m [13–15 ft] depth to groundwater [27]). The buildings in Fairbanks are spread over a large area of 28,000 m^2^ (300,000 ft^2^), with a variety of land covers including roads, parking lots, and buildings and limited open soil or vegetated areas. The not-for-profit and tuxedo and tailor rental buildings are within 30 m (100 ft) of the former dry cleaner that is believed to be the primary source of the underlying plume. The importance of source strength and the lateral distance from vapor source to building slab have been reported by others as metrics for identifying significant VI impacts [35–39]. The results here suggest that these two metrics—source strength and proximity—may also be useful in determining SSSG sampling probe density.

### 4.2. Temporal Trends in Soil Gas Across Fairbanks Buildings and Meteorological Drivers.

The results of 15 months of periodic sampling at the Fairbanks site show common seasonal elements, with SSSG PCE concentrations declining from December 2020 to May 2021 and increasing in August/September 2021 (Figure 1). However, the degree of change in concentration over time appears to be greater at the buildings that are further removed from source areas (such as the residential-style office building and the church) and less prominent in the buildings close to the point of release (such as the not-for-profit and tailor/tuxedo business). Even in the locations that show the greatest degree of change in response to seasonal weather patterns, the change was generally less than 1 order of magnitude; thus, somewhat less than the variability measured in SSSG at residential sites [2, 3, 40]. SSSG variability is generally less than indoor air variability at the same site [2, 3, 41].

The coefficient of variation (CV) is a way to describe the degree of variability within a data set that is independent of the absolute value. The CV is used here to directly compare the degree of variability of soil gas measurements at locations with varying concentration ranges. The temporal CV_t_ at individual points in the Fairbanks ranges from 0.15 to 2.67 for individual VOCs (Table 2). However, 32 of the 35 CV measurements fell in a narrower range of 0.31–1.87. In the Virginia Site A building (Table 3), the CV ranged from 0.83 to 2.31, with 10 of 11 CV_t_ values falling in a narrower range of 0.83–1.85. These estimates of CV_t_ from sites intensively studied with high-frequency sampling over more than a year could be used to estimate uncertainty for sparser sampling designs at other sites with similar CSMs and geology, as we discuss below.

### 4.3. Temporal and Spatial Variabilities in Fairbanks and Virginia SSSG Ports.

**Figure 3:**
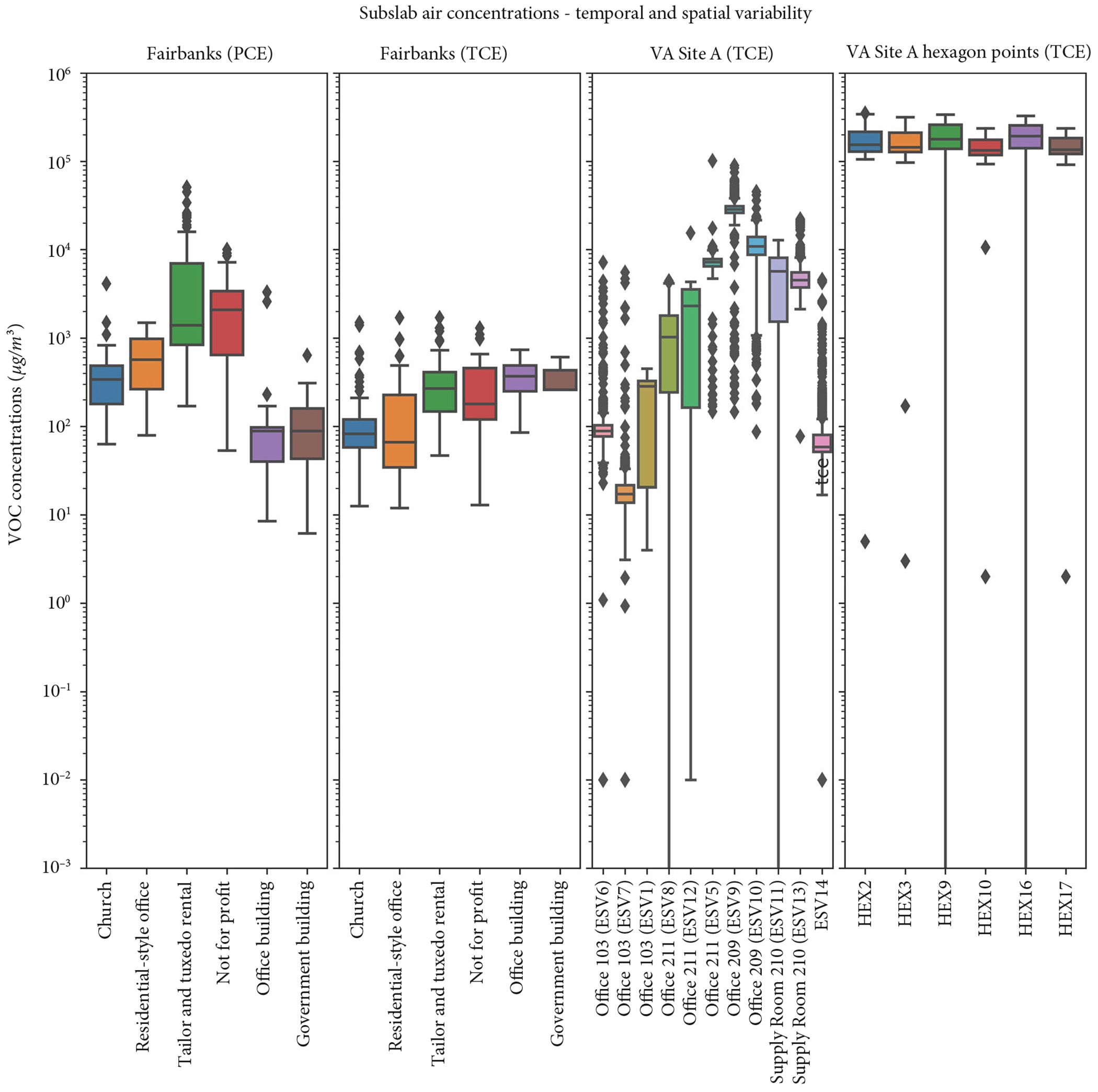
Box-and-whisker plots for SSSG PCE and TCE concentrations across sites and buildings or sample locations monitored for 12 months or longer. The boxes represent the 25th and 75th percentile values, the middle line is the median, the whiskers are the quartile ±1.5 times the interquartile range, and the plotted points are outliers. Results from the Fairbanks site include 5041 samples collected from December 1, 2020, to March 15, 2022. The number of samples and period of collection from Virginia Site A varies by location between 693 and 4849 samples collected from May 17, 2019, to June 2, 2021.

The spatial variability of PCE between mean concentrations measured during the sampling period varies by a factor of six or less across SSSG ports for all the individual building sites in Fairbanks except the tuxedo and tailor shop where the variability across points is a factor of 14 (Table 3 and Figures 3 and 2). In contrast, the concentration among the SSSG ports in a larger building at Virginia Site A varied by a factor of 1285 across a set of locations as described previously (Table 2 in Zimmerman et al. [28]). The spatial variability results suggest that large buildings above or very near a release had more spatial variability than buildings adjacent to or downgradient from release sites. This finding is consistent with and is reflected in the state regulatory guidance for the number of SSSG points per building, which recognizes the role of discrete release points as well as the variety of construction types within some larger buildings [8, 41].

### 4.4. Estimation of the Number of SSSG Samples to Collect to Characterize VI in a Large Building.

EPA VI guidance recommends collecting samples at multiple locations then, “The individual sample concentrations may be compared to the appropriate medium-specific screening levels” [1]. The individual media and location data are then put into context using a MLE approach which recognizes that SSSG is spatially variable and that any given indoor environment is influenced by soil gas concentrations. A formal risk assessment is then performed using either the maximum soil gas concentration or the 95th upper confidence limit on the mean soil gas concentration. In formal risk assessment, SSSG data is used to predict future risk at the existing buildings.

EPA’s *Guidance on Systematic Planning Using the Data Quality Objectives* Process [41] can be used to calculate a statistically defensible SSSG sample density for a larger building. The DQO guidance states: “The process of determining a minimum sample size relies on an estimate of total variability in the data to be collected. Sources of information on this estimate could include a pilot study of the same population, another study conducted with a similar population…” Hence, the user could apply the data from this study as a “similar population” from which to provide estimates of variability to be expected in SSSG. The estimation of the number of SSSG samples to collect in each building is analogous to the example for household lead sampling provided as Section 9.3 of the EPA systematic planning guidance document if the decision unit is the subslab concentration below the building as a whole [42]. The relevant determination of the number of SSSG samples (*n*) for a given building is

$$n=\frac{{\left(Z_{1-\alpha }+Z_{1-\beta }\right)}^{2}\sigma^{2}}{{\left(\bar{X}-AL\right)}^{2}}+\frac{1}{2}{Z_{1-\alpha }}^{2}$$

where $Z_{\left(1-\alpha \right)}=1.6449$, $Z_{\left(1-\beta \right)}=0.84$, σ = standard deviation of the concentrations of the building SSSG samples, $\bar{X}$ = mean of concentrations of a building SSSG samples, AL = action Level, *n* = number of samples, α is the false positive or Type I error rate, and β is the false negative or Type II error rate.

Note that in this equation, the number of required samples increases with increasing variability of the measurements. The number of samples is inversely proportional to the square of the difference between the mean concentration and the action level. The results of the application of this example to the data from Fairbanks and Virginia sites are presented in Table 4.

At four of the Alaska locations (church, office, residential office, and not for profit), the number of SSSG samples estimated from the TCE data varied between 5 and 26 times the number of SSSG samples estimated from the PCE data for the same building. The Alaska DEC action level for TCE (84 *μ*g/m^3^) is 21 times lower than the action level for PCE (1800 *μ*g/m^3^), and the average CV for TCE is greater than for PCE at four of the five buildings, which could account for some of the difference in calculated numbers. This is because the number of samples calculated by the equation is high when the mean of the data closely approaches or exceeds the action level of the compound of interest, which is the case for TCE in all of the buildings where it was detected and PCE in the tuxedo and tailor shop and the not-for-profit buildings in Alaska. In cases where the estimated sample requirement is impractically high, a screening study with a smaller number of samples may be necessary to refine the estimate of the average. If the estimate of the average is shown by the screening study to be very close to the action level, pre-emptive mitigation of at least part of the building or collection of indoor air data may be less costly than an initial collection of a very large number of subslab samples. The estimated number of SSSG samples for PCE and TCE at one of the Alaska buildings, the not for profit, is identical. The difference in the number of calculated SSSG samples for two Alaskan businesses, tailor shop and not for profit, is interesting because these businesses are sharing adjacent portions of a larger slab of a strip mall comprised of four businesses directly adjacent to a known source of contamination, the defunct Coin King Laundromat (Figure S1b). The result of their close proximity to this known source results in mean concentrations above the action levels for PCE and TCE. Note also that in those cases that if a conventional uncertainty estimate of even ±1 standard deviation is applied to the mean, the uncertainty range encompasses the action level. Thus, the formula is indicating that if the subslab action level is treated as a “bright line,” that is, a threshold for mitigating or not mitigating, more samples are required to determine if the true mean subslab concentration below the structure is above or below the action level. The calculated numbers generated in Table 4 using the data for TCE or PCE should only be used as a minimum number, and the actual number of SSSG samples to collect at the site should also consider features and conditions of the building (e.g., past usage, separate foundations, and footers) or site (e.g., proximity to source and depth to groundwater) and the number of SSSG ports installed.

Guidance on determination of the initial number of SSSG ports to install at a building is provided in multiple state VI guidance documents [6–8, 43]. Specifically, the New Jersey VI Guidance (NJVIG) document [8] not only suggests an estimate of the minimum number of SSSG ports for a given square footage of the footprint of the building (Table 4) but also discusses features and conditions of the building or site that may increase the number of SSSG ports that may be needed at a specific building/site. For example, if the NJVIG document is applied to the buildings at the Alaska and Virginia sites, four of the Alaska site buildings would result in two SSSG ports: one with a result of three SSSG ports and two which would require six. These numbers would be a more realistic number of initial SSSG ports for site investigation but could be modified to consider building or site features or conditions. Future additional SSSG ports should be added to each building location to better delineate the plume of the different contaminants detected (i.e., TCE and PCE) at a site.

Initial site evaluation data sets of individual buildings or residences will not include data comprised of several seasons of varied temporal samples with which to calculate a robust number of initial SSSG samples to collect. The data from this study can be utilized to calculate an estimate of initial SSSG samples to collect in a building at a new site. The use of this multiseason data set, to generate initial numbers of SSSG samples to install at a different site, would account for temporal and spatial variabilities over several seasons from a building in a similar climate zone. The calculated number of SSSG samples would provide increased confidence in the data collected for the new building or site investigation, sufficient to evaluate indoor air risk and impact risk management decisions for potential VI issues.

## 5. Conclusions

The use of statistical calculations, such as those described in Section 9.3 of the EPA systematic planning guidance document [42], is presented as an example of estimating the number of SSSG samples to collect in a building to achieve a selected confidence level; however, one should also be aware of site- and building-specific features and conditions, such as those discussed here and mentioned in the NJVIG [8] (e.g., presence of earthen or damaged floors, presence of cracked or damaged basement walls, presence of crawl-spaces, preferential pathways, and portion of building overlying or contacting the highest levels of VOC) that might require modification to the calculated number of SSSG samples. In this paper, we have presented an analysis of the factors controlling the observed spatial and temporal variabilities in a set of commercial buildings drawn from two sites in very different climate zones. The estimated sample requirement ranged from 1 to 80 samples; thus, it showed the substantial sensitivity of the systematic project planning equation to cases in which the action level and the average concentration are similar. The role of proximity to the point of release and seasonal meteorological factors in controlling spatial and temporal variability has been illustrated. Key descriptive statistics, including the range and CV from these data sets, have been tabulated, and the CVs are shown to be similar for the two sites. The ways this information can be applied to the systematic planning process for future building investigations have been illustrated with reference to the conceptual frameworks provided in EPA guidance documents.

## Supplementary Material

Supplement1

Additional supporting information can be found online in the Supporting Information section. (Supporting Information) The following supporting information can be downloaded at http://www.xxxxx.com/xxx/s1. Figure S1a: Eastern and western plumes close to their sources, Fairbanks, Alaska: (a) dry cleaner, (b) a not-for-profit organization, (c) a tuxedo and tailor rental shop, and (d) a not-for-profit office. Figure S1b: Historical sampling results and other site information. Figure S2a: Church basement sampling locations. Figure S2b: Church second floor sampling locations. Figure S3a: Residential-style office building basement sample locations. Figure S3b: Residential-style office building first floor sample locations. Figure S4: Location of subslab ports in the tailor and tuxedo rental property. Figure S5: Location of subslab ports in the nonprofit building. Figure S6: Subslab sampling locations in the large government-building basement. Figure S7: Subslab port locations in the office building. Figure S8: Monthly wind roses from Fairbanks International Airport during the project period. Figure S9: Soil temperature measured at the church. Table S1: Historical sampling results from Fairbanks buildings. Table S2: Building and HVAC characteristics. Table S3: Sample locations at Fairbanks and Virginia Site A buildings. References [17, 18, 20, 23–29, 31, 35, 40–42, 45–50] are cited in the supporting information.

## Figures and Tables

**TABLE 1:** Building characteristics.

Building	Site	Building characteristics				Date of construction, additions, and major renovations	Number of SSSG points
		Description	Approximate area	Number of stories	General construction material/style		
Not for profit	Fairbanks	Nonprofit organization; fourplex	369 m^2^ (3975 ft^2^)	1	Cinder block (property tax site says 20 cm [8″] concrete block)	1950	3
Tailor and tuxedo rental	Fairbanks	Tailor and tuxedo rental business; fourplex	454 m^2^ (4890 ft^2^)	1	Cinder block	1950	3
Residential-style office	Fairbanks	Residential-style building; business office	149 m^2^ (1600 ft^2^)	2 and basement	Wood frame over poured concrete	1951	2
Government building	Fairbanks	Large Government Building with basement	6503 m^2^ (70,000 ft^2^)	2 and basement	Primarily reinforced concrete	Constructed in 1933–1934 with additions in 1939 and 1948. Some renovations in 1994	3
Real estate building	Fairbanks	First floor real estate office Second floor real estate office	156 m^2^ (1680 ft^2^)	2	Concrete slab; brick and wood frame	1970	2
Church	Fairbanks	Place of worship	557 m^2^ (6000 ft^2^)	Split-level first floor over basement	Cinder block walls in basement; wood frame above	1952	3
Large industrial building	Virginia Site A	Warehouse-type building used for storage, maintenance, and administrative activities with office spaces, break rooms, restrooms, a library, and a mechanical room	11,200 m^2^ (120,400 ft^2^)	1	Brick exterior, wood and steel support structure, and a poured concrete slab generally 15–20 cm (6–8″) thick	1943	20

**TABLE 2:** Descriptive statistics of SSSG concentrations at Alaska site, by building, location, and analyte (all data in micrograms per cubic meter).

Building	Location	Analyte	Q_95	Q_90	Q_75	Q_50	Q_25	Q_10	Q_5	Mean	Count	Std_dev	CV (RSD)
Church	F1, main Rm, central	PCE	676	612	490	231	170	107	231	387	49	575	1.49
Church	F1, storage Rm, NW	PCE	495	470	318	170	133	112	170	231	47	139	0.60
Church	F1, storage Rm, SE	PCE	696	644	550	440	364	259	440	467	50	206	0.44
Church	F1, storage Rm, NW	cis-1,2-DCE	1560	1420	1000	820	86	84	820	738	5	681	0.92
Church	F1, storage Rm, SE	cis-1,2-DCE	1500	1400	1100	930	85	81	930	759	5	665	0.88
Church	F1, main Rm, central	TCE	409	332	163	83	61	55	83	151	18	164	1.08
Church	F1, storage Rm, NW	TCE	421	297	165	68	43	34	68	142	15	171	1.20
Church	F1, storage Rm, SE	TCE	520	244	125	94	71	56	94	172	25	278	1.61
Large government building	F1, BG club teen center, NW	PCE	154	148	131	102	72	55	102	102	2	83	0.82
Large government building	F1, gym, central	PCE	152	145	122	84	62	48	84	94	3	61	0.65
Office building	F1, east Office 2	PCE	852	398	100	69	42	15	69	207	9	416	2.01
Office building	F1, mech room	PCE	1725	150	98	94	42	31	94	364	11	974	2.67
Office building	F1, east Office 2	cis-1,2-DCE	1280	1260	1200	1100	84	83	1100	753	5	616	0.82
Office building	F1, east Office 2	TCE	678	615	460	325	175	113	325	351	6	241	0.69
Office building	F1, mech room	TCE	656	592	400	260	250	238	260	372	5	206	0.55
Residential-style office	B, central closet	PCE	472	388	319	250	180	144	250	262	53	106	0.40
Residential-style office	B, east, bottom of stairs	PCE	1300	1235	1100	970	680	532	970	896	54	278	0.31
Residential-style office	B, central closet	cis-1,2-DCE	1334	1258	1030	890	120	99	890	707	5	584	0.83
Residential-style office	B, central closet	TCE	686	449	193	61	25	17	61	176	14	270	1.54
Residential-style office	B, east, bottom of stairs	TCE	696	546	230	68	44	33	68	185	17	264	1.43
Not for profit	Conference Rm, NW	PCE	5300	4650	3475	2400	1700	1230	2400	2658	44	1345	0.51
Not for profit	Dining Rm, E	PCE	2700	750	300	240	190	170	240	559	31	1045	1.87
Not for profit	Mech Rm, SE	PCE	7990	6120	4350	3000	2200	846	3000	3492	47	2291	0.66
Not for profit	Conference Rm, NW	cis-1,2-DCE	1168	1136	1040	880	530	320	880	753	3	522	0.69
Not for profit	Dining Rm, E	cis-1,2-DCE	945	900	810	510	180	170	510	527	6	382	0.73
Not for profit	Conference Rm, NW	TCE	748	446	215	165	150	123	165	270	12	280	1.04
Not for profit	Dining Rm, E	TCE	834	688	250	210	170	170	210	356	5	350	0.98
Not for profit	Mech Rm, SE	TCE	655	310	245	180	33	30	180	213	11	282	1.32
Tuxedo and tailor shop	Closet, central	PCE	2900	2560	1850	1200	775	510	1200	1585	47	1716	1.08
Tuxedo and tailor shop	Front desk, N	PCE	1835	1400	1200	880	650	422	880	948	47	440	0.46
Tuxedo and tailor shop	Storage Rm, S	PCE	41500	27700	15250	9700	7100	4380	9700	13604	48	11381	0.84
Tuxedo and tailor shop	Front desk, N	cis-1,2-DCE	1083	1066	1015	930	870	834	930	947	3	146	0.15
Tuxedo and tailor shop	Closet, central	TCE	842	530	305	145	77	66	145	273	12	327	1.20
Tuxedo and tailor shop	Front desk, N	TCE	371	333	173	106	91	71	106	159	8	127	0.80
Tuxedo and tailor shop	Storage Rm, S	TCE	1300	1240	580	350	210	170	350	494	37	408	0.83

*Note:* “Q_#” represents quartile, that is, Q_95 represents 95th quartile. N, S, E, W, SE, and NW represent abbreviations of directions.

Abbreviations: B, basement; cis-1,2-DCE, cis-1,2-dichloroethene; CV, temporal coefficient of variation; F1, Floor 1; PCE, tetrachloroethylene; RSD, relative standard deviation; TCE, trichloroethylene.

**TABLE 3:** Descriptive statistics of Virginia Site A SSSG concentrations, by building, location, and analyte (all data in micrograms per cubic meter).

Site	Building	Location/floor/room	Instrument/method	Sample type and duration	Analyte	Q_90	Q_50	Q_10	Mean	Count	Std Dev	CV (RSD)
Virginia Site A	Large industrial building	Women’s restroom	GC	IA daily	PCE (*μ*g/m^3^)	2.32	0.23	0.09	0.91	589	2.09	2.31
Virginia Site A	Large industrial building	Women’s restroom	GC	IA weekly	PCE (*μ*g/m^3^)	2.42	0.36	0.11	0.90	87	1.22	1.35
Virginia Site A	Large industrial building	Office adjacent to restroom	GC	IA daily	PCE (*μ*g/m^3^)	3.46	0.50	0.50	1.26	230	1.52	1.20
Virginia Site A	Large industrial building	Office adjacent to restroom	GC	IA weekly	PCE (*μ*g/m^3^)	2.80	0.82	0.50	1.24	35	1.03	0.83
Virginia Site A	Large industrial building	Supply room	GC	IA daily	PCE (*μ*g/m^3^)	2.31	0.55	0.17	0.96	589	1.07	1.11
Virginia Site A	Large industrial building	Supply room	GC	IA weekly	PCE (*μ*g/m^3^)	2.19	0.62	0.18	0.96	87	0.83	0.86

*Note:* “Q_#” represents quartile, that is, Q_95 represents 95th quartile.

Abbreviations: GC, gas chromatograph; IA, indoor air; PCE, tetrachloroethylene; RSD, relative standard deviation.

**TABLE 4:** Results of the application of relevant equations to determine SSSG samples needed to characterize VI in a large building.

State	Building	Location	Analyte	Mean (*μ*g/m ^3^)	Number of samples	Standard deviation	State commercial action level	EPA QA/G4 estimated sample size–state commercial action level
Alaska	Church	Entire building	PCE	355	224	273	1800	2
Alaska	Church	Entire building	TCE	155	58	204	84	52
Alaska	Office building	Entire building	PCE	286	20	695	1800	3
Alaska	Office building	Entire building	TCE	336	11	381	84	15
Alaska	Residential-style office	Entire building	PCE	579	107	192	1800	2
Alaska	Residential-style office	Entire building	TCE	181	31	267	84	48
Alaska	Not for profit	Entire building	PCE	2236	122	1560	1800	80
Alaska	Not for profit	Entire building	TCE	280	28	304	84	16
Alaska	Tailor and tuxedo shop	Entire building	PCE	5379	142	4512	1800	11
Alaska	Tailor and tuxedo shop	Entire building	TCE	309	57	287	84	11
Alaska	Large government building	Entire building	PCE	171	8	198	1800	1
Virginia	Large industrial building	Entire building	TCE	6978	36,916	10,086	2400	31

## Data Availability

The data that support the findings of this study are openly available in Data.gov at 10.23719/1532422.
