# Supplementary material for: Estimation of the Number of Subslab Soil Gas Samples to Collect to Characterize Vapor Intrusion Under a Large Building
Source: Indoor Air. Author manuscript; Available in PMC 2026 Aug 19. (PMC12425155; doi:10.1155/ina/2860696)
Supplement: Supplementary file 1 [file NIHMS2107042-supplement-Supplement1.docx]

Estimation of the Number of Soil Gas Samples to Collect to Characterize Vapor Intrusion Under a Large Building

John Zimmerman,^a*^ Alan Williams,^a^ Brian Schumacher,^b^ Christopher C. Lutes,^c^ Rohit Warrier^g^, Laurent Levy,^d^ Gwen Buckley,^e^ Brian Cosky,^f^ Chase Holton,^h^ and Kate Bronstein^g^

a. U.S. Environmental Protection Agency (EPA), Office of Research and Development (ORD), Center for Environmental Measurement & Modeling (CEMM), Watershed and Ecosystem Characterization Division (WECD), 109 T.W. Alexander Drive, P.O. Box 12055, Mail Drop: D205-7, RTP, NC, 27711, USA
b. U.S. EPA, ORD, CEMM, Ecosystem Processes Division, 960 College Station Road, Athens, GA, 30605, USA
c. Federal and Environmental Solutions, Jacobs Engineering Group Inc., 111 Corning Road, Suite 200, Cary, NC, 27518, USA
d. Federal and Environmental Solutions, Jacobs Engineering Group Inc., 120 St. James Avenue, 5th Floor, Boston, MA, 02116, USA
e. Federal and Environmental Solutions, Jacobs Engineering Group Inc., 5701 Cleveland St, Suite 200, Virginia Beach, VA, 23462, USA
f. Federal and Environmental Solutions, Jacobs Engineering Group Inc., 1099 North Meridian Street, 5th Floor, Indianapolis, IN, 46204, USA
g. Center for Environmental Health, Risk, and Sustainability, RTI International, 3040 E. Cornwallis Rd., Research Triangle Park, NC, 27709, USA
h. Denver Office, GSI Environmental Inc., 13949 W. Colfax, No. 210, Lakewood, CO, 80401, USA

* Corresponding Author: John H. Zimmerman, zimmerman.johnh@epa.gov, USEPA/ORD/CEMM/WECD, 109 T.W. Alexander Drive, P.O. Box 12055, Mail Drop: D205-7, RTP, NC, 27711, USA, (919) 541-9719

**Supplemental Information**

The supplemental information provided below contains further details and supporting data to accompany the main research article, including discussion of sources and conceptual site models, site regional geology and climatology, additional details of buildings and their HVAC characteristics, building floor plans and sampling locations, site weather, additional site figures and data tables. These items are intended to enhance the reader's understanding of the study's findings and allow for greater transparency in the research process.

**Sources and Conceptual Site Models**

**Sources and conceptual site models—eastern plume**

The eastern plume is thought to originate from a laundromat that has been operational since at least 1960 and did dry cleaning on site until 1998, when it transitioned to providing drop-off services for off-site dry cleaning work and on-site coin-operated laundry operations.^1^ This dry cleaner is in a “four-plex” of abutting or nearly abutting buildings separated by alleys facing Gaffney Road (Figure S1a, S1b). The four-plex consists of the (a) dry cleaner, (b) a Not-for-profit organization, (c) a tuxedo and tailor rental shop, and (d) a not-for-profit office.

Several multimedia investigations have been performed to characterize the source, nature, and extent of release (e.g., reports from Oasis Environmental^2-4^). A second dry cleaner lies approximately 45.7 meters (m) (150 feet [ft]) to the east.

In general, the highest soil contaminant concentrations identified were near the southeast corner of the former dry cleaner building, where the historic presence of a drum storage area was noted.^5^ Tetrachloroethylene (PCE) was detected in soil samples collected between 0 and 4.6 m (0 and 15 ft) below ground surface, with a maximum PCE concentration of 82,000 micrograms per kilogram (µg/Kg) collected from boreholes on the south side of the dry cleaner building. The soil source area extends from the back of the building out under the pavement on Airport Way.^5^ As in the soil, elevated PCE concentrations in groundwater have been reported near the former drum storage area south of the dry cleaner building, with a maximum PCE concentration of 4,100 micrograms per liter (µg/L). As of 2014, a detectable groundwater solvent plume extends from this area northwest to 10th Avenue (Figure S1b). Statistical analyses suggest that the groundwater plume is stable.^4^

Historical vapor intrusion (VI) assessments have been conducted at several buildings discussed in this study. Table S1 summarizes VI samples historically reported at each building. The residential-style office was believed to have been affected by the eastern plume through a primarily sewer transport mechanism and was included as part of this study (Figure S1b). This building has a basement approximately 1.8 m (6 ft) deep,^6^ an attached former garage, and two above-ground stories. The building is a bungalow- or cape-style construction^7^ and was built in 1951 with a wood frame.^8^ This building was sampled between 2008 and 2011, with maximum subslab concentrations of 19,000 µg/m^3^ (PCE) and 860 µg/m^3^ (TCE); maximum indoor air concentrations were 4.3 µg/m^3^ (PCE) and 0.19 µg/m^3^ (TCE) (Table S1; Weller, personal communication). The VI pathway for this building was determined to be complete, although PCE concentrations in the workspace at the time of sampling were below Alaska Department of Environmental Conservation (ADEC) target levels.^9^

**Sources and CSM—western plume**

The western plume is thought to have originated from releases at or from the sewer service line at a former dry cleaning service site and a former auto service station located on Gaffney Road between Noble and Turner Streets.^5^ The eastern plume may also have contributed to the western plume through wood stave sewer lines.^10^ Investigative, remedial, and monitoring work has occurred at Gaffney West since 1997.^4^ Three sources of PCE-contaminated soil were identified beneath and adjacent to the former Launderette building at the location of MW-9 between 1.5 and 3.7 m (5 and 12 ft) below ground surface,^11^ which was later occupied by a bookstore (on Gaffney Road, west of the four-plex). During a 2004 source characterization study, a wood stave secondary sanitary sewer line for the bookstore was breached, and 6.9 m^3^ (9 cubic yards) of PCE-affected soil were removed. The western groundwater plume extends approximately 914 m (3,000 ft) from the Airport Way/Cushman Street intersection in a northwest direction toward the Chena River (Figure S1a). VI assessments were completed at several buildings overlying the western plume (Table S1). A long-term study of the bookstore is reported in Barnes and McRae.^12^

A soil vapor extraction/subslab depressurization system was installed in 2010 to mitigate the VI in the bookstore building. The system operated intermittently until 2018, when it was converted to a subslab depressurization system, only with a much smaller blower. Soil gas data showing concentrations of PCE up to 7,800 µg/m^3^ in external soil gas in 2018 show that there is still a strong residual source present, which is likely to continue to support the western plume.^13^

The church also overlies the western plume and was historically sampled for VI (Figure S1b and Table S1). The church had a maximum subslab PCE concentration of 1,700 µg/m^3^ and a maximum indoor air concentration of 0.68 µg/m^3^ in the single round of previous sampling in 2007. Groundwater at the aforementioned locations is shallow, with depth to water at around 4.6 m (15 ft) below ground surface.^4^

**Figure S1a. Eastern and western plumes close to their sources, Fairbanks, Alaska^45^: a) dry cleaner, b) a Not-for-profit organization, (c) a tuxedo and tailor rental shop, and (d) a not-for-profit office.**

**Figure S1b. Historical sampling results and other site information. Reprinted from Ahtna Environmental, Inc.8**

| **Business** | **Time Period** | **Sampling Events (#)** | **Events per Season (#)** | **Types and Number of Samples** | **Max PCE Concentration (µg/m^3^)** | **Median PCE Concentration (µg/m^3^)** | **Max TCE Concentration (µg/m^3^)** | **Median TCE Concentration (µg/m^3^)** |
| --- | --- | --- | --- | --- | --- | --- | --- | --- |
| Dry cleaner | 2013 | 1 | Winter: 0 | Subslab: |  |  |  |  |
|  |  |  | Spring: 0 | Indoor: 3 | 20 | 20 | 7 | 4.3 |
|  |  |  | Summer: 0 | Long-term passive: |  |  |  |  |
|  |  |  | Fall: 1 | Crawl space: |  |  |  |  |
|  |  |  |  | Exterior soil gas: 2 | ND (0.23) | ND (0.21) | ND (0.18) | ND (0.16) |
| Residential style office building | 2008–2011 | 9 | Winter: 3 | Subslab: 18 | 19,000 | 7,300 | 860 | 335 |
|  |  |  | Spring: 2 | Indoor: 11 | 4.3 | 1.7 | 0.19 | ND (0.18) |
|  |  |  | Summer: 1 | Long-term passive: 0 | -- | -- | -- | -- |
|  |  |  | Fall: 3 | Crawl space: 0 | -- | -- | -- | -- |
|  |  |  |  | Exterior soil gas: 2 | 5,900 | 5,150 | 400 | 355 |
| Church | 2007 | 1 | Winter: 0 | Subslab: 3 | 1,700 | 34 | ND (2.6) | ND (2.6) |
|  |  |  | Spring: 0 | Indoor: 1 | 0.68 | 0.68 | ND (0.14) | ND (0.14) |
|  |  |  | Summer: 1 | Long-term passive: 0 | -- | -- | -- | -- |
|  |  |  | Fall: 0 | Crawl space: 0 | -- | -- | -- | -- |
|  |  |  |  | Exterior soil gas: 0 | -- | -- | -- | -- |
|  |  |  | Fall: 2 | Crawl space: 0 | -- | -- | -- | -- |
|  |  |  |  | Exterior soil gas: 2 | 9,500 | 5,700 | 150 | ND (75) |
| Tailor and tuxedo rental business | 2009–2011 | 6 | Winter: 2 | Subslab: 14 | 44,000 | 8,950 | 1,500 | 200 |
|  |  |  | Spring: 1 | Indoor: 7 | 8,500 | 68 | 140 | 2 |
|  |  |  | Summer: 1 | Long-term passive: 0 | -- | -- | -- | -- |
|  |  |  | Fall: 2 | Crawl space: 0 | -- | -- | -- | -- |
|  |  |  |  | Exterior soil gas: 4 | 29,000 | 11,100 | 2,000 | 260 |
| Not-for-profit | 2009–2014 | 7 | Winter: 2 | Subslab: 14 | 17,000 | 970 | 85 | 9.45 |
|  |  |  | Spring: 1 | Indoor: 8 | 41 | 18.5 | 0.61 | 0.53 |
|  |  |  | Summer: 2 | Long-term passive: 2 | 11 | 10.05 | 0.16 | 0.135 |
|  |  |  | Fall: 2 | Crawl space: 0 | -- | -- | -- | -- |
|  |  |  |  | Exterior soil gas: 2 | 1,600 | 1,055 | 64 | ND (32) |

**Table S1. Historical sampling results from Fairbanks buildings**

**Regional Geology and Climatology**

Absolute maximum daily temperatures up to 36°C (97°F) have been recorded in June, and minimum daily temperatures up to −54°C (-129°F) have been reported in January at Fairbanks.^14^ Maximum and minimum hourly air temperatures of 32°C (89°F) to -43°C (-45°F) were recorded during this study period (December 1, 2020 to April 1, 2022) at the Fairbanks International Airport (PAFA). Compared with some other regions in Alaska, Fairbanks receives lower annual precipitation because it is sheltered by the Alaska Range and Brooks Range to the south and north, respectively. Mean annual precipitation for the period from 1916 to 2006 was only 28 centimetres (cm) (11 inches[in]), with most of the precipitation observed in the summer.^14^ In the winter, monthly average snowfall between 23 and 30.5 cm (9 to 12 in) is observed between November and March, with maximum daily average snow depths of up to 51 cm in February.^14^ During the winter, Fairbanks routinely experiences strong temperature inversions (i.e., temperature increases with elevation) owing to lack of wind, a snow-covered surface, and insufficient sunlight to heat the ground. There are known to be substantial localized variations in weather in the Fairbanks area itself based on elevation and local topography, sometimes as much as 28°C (82°F) during inversions.^14^ These inversions can trap pollutants from burning of wood, fuels, and automobiles, including fine particulate matter smaller than 2.5 microns (PM_2.5_), leading to poor air quality.^18^

The study site is situated on the collective floodplain of the Tanana and Chena Rivers. The surficial geology consists of well-stratified layers of unconsolidated silt, sand, and gravel of the Chena alluvium, which is approximately 91 m (299 ft) thick in river valleys.^18^ Test borings conducted in the vadose zone at the site confirmed the presence of alternating layers of sand with varying gravel and silt compositions with the interval near the top of the water table (4.6 m to 5.2 m [15 ft to 17 ft] below ground surface) characterized as poorly graded gravel with sand.^12^ Discontinuous permafrost of generally low ice content is characteristic of Chena alluvium sediments, but permafrost in the form of seams and lenses maybe found in swale and slough deposits. Where present, permafrost ranges in depth from 0.6 m to 12.2 m (2 ft to 40 ft) below ground surface.^18^ The geology underlying the site may yield some radon potential. Fairbanks is in EPA Radon Zone 2,^20^ and a 16 km^2^ hexagon around the site has historical radon observations between 0 and 34 picocuries per liter (pCi/L), with a mean of 2.57 pCi/L based on 45 observations.^21^

The unconfined, alluvial-plain Chena alluvium aquifer can yield significant quantities of water in wells. Recharge to the alluvial-plain aquifer occurs from the Tanana and Chena Rivers, with a relatively small amount resulting from infiltration of precipitation. Groundwater levels in the alluvial-plain aquifer respond relatively quickly to increases in the stages of the Tanana and Chena Rivers. Wells completed in the alluvial-plain aquifer within half a mile of either river show the greatest elevation increases because of increased river flow.^22^ Data gathered during previous groundwater assessments at Gaffney Road East and Gaffney Road West indicate that groundwater in the unconfined alluvial-plain aquifer is shallow at about 6 m (20 ft) below ground surface, with flow generally toward the northwest.^20^

Building and HVAC characteristics

Significant changes in the routine position or settings of interior and exterior doors, windows, and heating, ventilation, and air conditioning (HVAC) systems were documented on data collection forms and summarized in an event log (not shown).

Larger commercial structures can have many HVAC and sampling zones (offices, factory floor, etc.). HVAC zones (thermal zones) are spaces controlled with one thermostat typically served by a single air handling unit (see Table S2). Sample zones are enclosed locations in a building where at least one indoor air sample has been collected. The HVAC or sample zones of primary interest to this study were those nearest the points of entry of soil gas into the building. Therefore, this study focused primarily on the basement (if present) and first floor. Additional zones in the studied buildings were identified through a visual and interview-based survey process.

The Church is a 557 m^2^ (6,000 ft^2^), two-story building with a basement (Figure S2a and S2b). Entry to the building is through a first-floor split-level with stairs leading to the basement and second floor. The presence of a basement sump and significant cracks were noted in the basement slab at the time of the initial survey. The building is primarily heated through hot water radiators supplied from the city’s central hot water system. No known cooling systems were noted in the building but bathroom vents with outside air intake were documented.

The Residential-style office is a 149 m^2^ (1,600 ft^2^), two-story residential-style building with a basement and attached garage on the east side of the building. The building is primarily used as a Residential-style office with several offices, a bathroom, kitchen and breakroom located on the first floor and more office and storage rooms on the second floor (Figures S3a and S3b). The concrete-floored basement is accessed through the former attached garage and is primarily used as a storage space and includes a closet and a boiler. The boiler provides hot water to radiators at baseboards for heating the building. Additional ventilation systems include fans in the bathrooms. No working cooling systems were documented.

The commercial tailor and tuxedo rental facility is also located within the four-plex. This 454 m2 (4,890 ft^2^), single-story structure includes a storeroom, tuxedo storage room, central closet, office, several bathrooms and an apartment with a bathroom and kitchen to the south of the building (Figure S4). The building is primarily heated through hot water baseboards generated by a boiler system with additional space heaters used in storage rooms. Although the building has access to central air cooling, it is not used. Additional ventilation systems include fans in storage rooms for in-room heating.

The Not-for-profit is a 369 m^2^ (3,975 square feet [ft^2^]), single-story commercial structure that is part of the four-plex and is primarily used for food services. The structure was constructed in 1950 with cinder blocks and consists of a conference room, kitchen, dining room, storage and bathrooms (Figure S5). The primary heating system for this building is through hot air circulated via ducts and generated by two boiler systems which heat water to heat air. The building is cooled via central air and has other ventilation systems such as a kitchen hood fan and bathroom fans. Based on the building survey, a single HVAC zone was determined for this structure. HVAC zones (thermal zones) are spaces controlled with one thermostat typically served by a single air handling unit.

Government building is a two-story, approximately 6500 m^2^ (70,000 ft^2^) government-building with a basement that is partially below-grade. The building has several offices on the first and second floors and a gym, teen center, storage/file rooms and a maintenance room with the main air unit located in the basement (Figure S6). The building is primarily heated through radiators receiving steam from the district steam plant. Building cooling is achieved through central air in some areas with most offices having individual AC units. Ventilation is achieved through large vents in most portions of the building from overhead or wall vents.

The office building is a two-story, 156 m^2^ (1,680 ft^2^) building that is primarily used as office space (Figure S7). This building is primarily heated through hot water radiators supplied by an onsite boiler and cooled using central air on the first floor with individual AC units used on the second floor. Additional ventilation systems include several bathroom fans on both floors.

| **Building** | **Building Characteristics** | | | |  | **HVAC Characteristics** | | | | | | | |
| --- | --- | --- | --- | --- | --- | --- | --- | --- | --- | --- | --- | --- | --- |
|  | **Description** | **Approximate Area in m^2^ (ft^2^)** | **Number of Stories** | **General Construction Material/Style** | **Date of Construction, Additions, and Major Renovations** | **Basement or Crawl Present?** | **Heating System** | **Heat Generation** | **Heat Distribution** | **Cooling System** | **Ventilation Systems** | **Number of Zones** |  |
| **Eastern Plume/Source Area** | | | | | | | | | | | | | |
| Not-for-profit | Nonprofit organization; four-plex | 369 (3,975) | 1 | Cinder block (property tax site says 8" concrete block) | 1950 | N | Hot air circulation | Boiler system appears to heat water to heat air; fuel oil used for 2 boilers | Air circulation ducts | Central air (inlet appears to be from outside) | Kitchen hood fan; bathroom fans | 1 |  |
| Tailor and tuxedo rental business | Tailor and tuxedo rentals; four-plex | 454 (4,890) | 1 | Cinder block | 1950 | N | Hot water baseboard (survey says electric baseboard) | Boiler system; fuel oil for boiler | Hot water to radiators at baseboards; space heater in storage rooms | Central air (not used) | Fans in storage rooms for in-room heating | 1 |  |
| Residential-style office | Residential-style building; business office | 149 (1,600) | 2 and basement | Wood frame over poured concrete | 1951 | Y, basement with separate access entrance through former garage on east side of building | Hot water radiation. Baseboards | Fuel oil for heating | Hot water to radiators at baseboards likely |  | Bathroom vents | 3 |  |
| Government building | Large government building with basement | 6500 (70,000) | 2 and basement | Primarily reinforced concrete | Constructed in 1933–34 with additions in 1939 and 1948. Some renovations in 1994. | Y, basement partially below grade | Hot water radiation | District steam plant | Radiators in most offices; air handling room on 3rd floor includes heated coils (steam) | (Survey says central air in some areas; most offices have individual AC units for use in summer) | Central ventilation with large vents in most portions of the building from overhead or wall vents |  |  |
| **Western Plume/Source Area** | | | | | | | | | | | | | |
| Office building | First-floor real estate office | 156 (1,680) | 2 | Concrete slab; brick and wood frame | 1970 | N | Hot water radiation | Fuel oil | Hot water to radiators at baseboards likely | First floor: central air, outside intake; second floor: individual AC units, possible summer use only; cool air not from outside | First floor: fans, bathroom fans; second floor: fans, bathroom fans | 3 |  |
|  | Second-floor real estate office |  |  |  |  |  |  |  |  |  |  |  |  |
| Church | Place of worship | 557 (6,000) | Split level first floor over basement | Cinder block walls in basement; wood frame above | 1952 | Y, basement partially below grade | Plate exchanger with hot water radiation (baseboards) | Hot water from city central hot water; fuel oil | Hot water to radiators at baseboards likely |  | Bathroom vents, outside air intake with exhaust at south end of chapel | Possibly 6 |  |

**Table S2. Building and HVAC characteristics**

Sampling and analysis

The indoor and outdoor air, subslab soil gas, and external soil gas monitoring locations in each building are summarized in Table S3. Sampling locations for all buildings in the Fairbanks study are shown in Figures S2 through S7.

**Figure S2a. Church basement sampling locations**

**Figure S2b. Church second floor sampling locations**

**Figure S3a. Residential-style Office building basement sample locations**

**Figure S3b. Residential-style Office building first floor sample locations**

**Figure S4. Location of subslab ports in the tailor and tuxedo rental property**

**Figure S5. Location of subslab ports in the nonprofit building**

**Figure S6. Subslab sampling locations in the large government building basement**

**Figure S7. Subslab port locations in the office building**

| **Building** | **Sample Type** | **Number of Sample Locations** | | **Sample Location within Building** | **Sample ID** |
| --- | --- | --- | --- | --- | --- |
| Residential-style office | Indoor air | 2 | | Floor 1, kitchen/office | IA-42 |
|  |  |  |  | Basement, central | IA-43 |
|  | Indoor air (GC) | 5 | | Basement 1 | IA-63 |
|  |  |  |  | Basement 2 | IA-65 |
|  |  |  |  | Garage | IA-66 |
|  |  |  |  | Floor 1, office | IA-67 |
|  |  |  |  | Floor 1, kitchen | IA-68 |
|  | Outdoor air (GC) | 2 | | Outside garage | OA-14 |
|  |  |  |  | OA-14 was relocated to OA-15 on 10/1/2021 | OA-15 |
|  | Subslab (GC) | 2 | | Basement, central closet | SS-59 |
|  |  |  |  | Basement, east, bottom of stairs | SS-60 |
|  | Radon | 2 | | Floor 1, kitchen/office | NA |
|  |  |  |  | Basement, main room | NA |
|  | Differential pressure | 2 | | Basement, main room | NA |
|  |  |  |  | Former garage | NA |
|  | Temperature | 1 | | Floor 1, kitchen/office | NA |
| Church | Outdoor air | | 1 | South end, away from building | OA-12 |
|  | Outdoor air (GC) | | 1 | Outside mechanical room vent | OA-13 |
|  | Indoor air | | 3 | B, main room, south end 1 | IA-50 |
|  |  |  |  | B, kitchen 1 | IA-51 |
|  |  |  |  | F2, chapel 1 | IA-52 |
|  | Indoor air (GC) | | 8 | Mechanical room | IA-57 |
|  |  |  |  | Mechanical room (moved from IA-57 on 2/9/2022 to opposite wall) | IA-69 |
|  |  |  |  | SE corner | IA-58 |
|  |  |  |  | Classroom | IA-59 |
|  |  |  |  | Entryway | IA-60 |
|  |  |  |  | Chapel corner | IA-61 |
|  |  |  |  | Chapel mid | IA-62 |
|  |  |  |  | Main room, south end | IA-50 |
|  | Subslab (GC) | | 3 | F1, main room, central | SS-54 |
|  |  |  |  | F1, storage room, NW | SS-55 |
|  |  |  |  | F1, storage room, SE | SS-56 |
|  | Soil gas | | 16 | West, 2' | SG-16 |
|  |  |  |  | West, 4.5' (GC location) | SG-17 |
|  |  |  |  | West, 7' (GC location) | SG-18 |
|  |  |  |  | West, 9.5' | SG-19 |
|  |  |  |  | East, 2' | SG-20 |
|  |  |  |  | East, 4.5' | SG-21 |
|  |  |  |  | East, 7' | SG-22 |
|  |  |  |  | East, 9.5' | SG-23 |
|  |  |  |  | South, 2' | SG-24 |
|  |  |  |  | South, 4.5' (GC location) | SG-25 |
|  |  |  |  | South, 7' (GC location) | SG-26 |
|  |  |  |  | South, 9.5' | SG-27 |
|  |  |  |  | North, 2' | SG-28 |
|  |  |  |  | North, 4.5' | SG-29 |
|  |  |  |  | North, 7' | SG-30 |
|  |  |  |  | North, 9.5' | SG-31 |
|  | Differential pressure | | 4 | Basement to floor 1 | NA |
|  |  |  |  | Mech room | NA |
|  |  |  |  | Central meeting room | NA |
|  |  |  |  | Auditorium hall | NA |
|  | Temperature | | 2 | Ambient | NA |
|  |  |  |  | Auditorium | NA |
|  | Radon | | 3 | Auditorium | NA |
|  |  |  |  | Central meeting room | NA |
|  |  |  |  | Ambient by Rad7 | NA |

| **Building** | **Sample Type** | **Number of Sample Locations** | **Sample Location within Building** | **Sample ID** |
| --- | --- | --- | --- | --- |
| Not-for-profit | Indoor Air | 2 | NW conference room | IA-46 |
|  |  |  | hallway adjacent to kitchen | IA-47 |
|  | Subslab | 3 | conference room. NW | SS-51 |
|  |  |  | mech room, SE | SS-53 |
|  |  |  | main central dining room | SS-52 |
|  | Differential pressure | 2 | near mech room, SE | NA |
|  |  |  | main central dining room | NA |
| Tailor and tuxedo rental business | Indoor Air | 2 | store room, central halfwall | IA-44 |
|  |  |  | storage room west, tuxedo room | IA-45 |
|  | Subslab | 3 | front desk, N | SS-48 |
|  |  |  | storage room, S | SS-49 |
|  |  |  | central closet | SS-50 |
|  | Groundwater | 2 | south of building | MW31 |
|  |  |  | north of building | MW33s |
|  | Radon | 2 | main front room | NA |
|  |  |  | west storage | NA |
|  | Differential pressure | 2 | main front room | NA |
|  |  |  | by back dock | NA |
|  | Temperature. | 1 | front entry room | NA |
| Government building | Indoor Air | 7 | Basement, B&G club teen center, kitchen counter1 | IA-35 |
|  |  |  | Basement, SW corner file room1 | IA-37 |
|  |  |  | Basement, SE corner storage room1 | IA-38 |
|  |  |  | Basement, NE corner, maintenance crew shop | IA-39 |
|  |  |  | Floor 1, B&G club gym, front desk | IA-36 |
|  |  |  | Floor 1, SW corner engineering technicians office equipment area | IA-40 |
|  |  |  | Floor 1, SE corner engineering office | IA-41 |
|  | Subslab | 3 | B, gym, central | SS-45 |
|  |  |  | B, SW file room | SS-47 |
|  |  |  | B, B&G club teen center, NW | SS-46 |
|  | Radon | 4 | Basement, B&G club teen center, kitchen counter | NA |
|  |  |  | Basement, SW corner file room | NA |
|  |  |  | Floor 1, B&G club gym, front desk | NA |
|  |  |  | Floor 1, SE corner engineering office | NA |
|  | Differential pressure | 2 | Basement, B&G club teen center, kitchen | NA |
|  |  |  | in southeast stairwell from basement to floor 1 | NA |
| Office Building | Indoor Air | 1 | F1, central reception on cabinet | IA-48 |
|  | Subslab | 2 | F1, east office 2 | SS-57 |
|  |  |  | F1, mech room | SS-58 |
|  | Radon |  | F1, main entrance | NA |
|  | Differential pressure | 2 | F1, boiler room | NA |
|  |  |  | stairwell floors 1 and 2 | NA |
|  | Indoor Air | 1 | F2, cabinet | IA-49 |
|  | Radon | 1 | F2, main entrance | NA |
| Virginia Site A | Subslab | 2 | Warehouse, Bay 1 and Bay 2 | ESV-1, ESV-5 |
|  |  | 2 | Office 103, West and East | ESV-6, ESV-7 |
|  |  | 2 | Office 211, Office and Breakroom | ESV-8, ESV-12 |
|  |  | 2 | Office 209, North and South | ESV-9, ESV-10 |
|  |  | 2 | Supple Room 210 | ESV-11, ESV-13 |
|  |  | 2 | Office 211 | ESV-14 |
|  |  | 6 | Bay 2 | HEX2, HEX3, HEX9, HEX10, HEX16, HEX17 |

**Table S3. Sample locations at Fairbanks and Virginia Site A buildings**

Weather

An on-site weather station (the RainWise MK-III system) used for this study recorded ambient temperature (T), relative humidity (RH), dewpoint T, wind speed and direction, barometric pressure, and rainfall. The airport weather station was more sophisticated compared with the on-site system and could measure frozen precipitation. The on-site weather station also experienced frequent outages because it shared computer network resources with the residential-style office. Results from the on-site system were compared with the airport’s weather station and generally showed good correlation. Monthly wind roses from Fairbanks International Airport during the project period (prepared on the Iowa State University Mesonet website; https://mesonet.agron.iastate.edu/ last accessed 9/30/2022) is shown in Figure S8. Soil temperatures measured during the project period at various depths in external soil gas probes at the Church are shown in Figure S9.

**Figure S8. Monthly wind roses from Fairbanks International Airport during the project period (prepared on the Iowa Environmental Mesonet of Iowa State University, website; https://mesonet.agron.iastate.edu/ last accessed 9/24/2024)**

**Figure S9. Soil temperature measured at the Church.**

**References**

45	Oasis Environmental, Source characterization report, Gaffney Road area, Fairbanks, Alaska, Anchorage, AK, 2008.
